# Microstructural Evolution in Solid-State Sn–Zn/Ag/Cu Interfacial Reactions at 170 °C

**DOI:** 10.3390/ma19194217

**Published:** 2026-10-03

**Authors:** Chao-Hong Wang, Chu-An Li

**Affiliations:** Department of Chemical Engineering, National Chung Cheng University, Chiayi 621301, Taiwan; contexzxc1101@gmail.com

**Keywords:** interfacial reaction, Sn-Zn, lead-free solders, Ag, surface finish

## Abstract

Ag is widely used as a surface-finish material in electronic packaging, while Sn–Zn is a representative lead-free solder system. This study investigates the interfacial reactions and microstructural evolution in Sn–Zn/Ag/Cu couples during solid-state aging at 170 °C. Unlike the conventional approach, in which Ag is electroplated onto Cu substrates followed by reflow soldering, Ag (~2.0 μm) and Cu (~50 μm) layers were sequentially electroplated onto three Sn–Zn solders containing 1, 3, and 9 wt.%Zn. By eliminating the reflow process, the Ag layer remained at the interface, allowing the reactions to proceed in the Sn–Zn/Ag/Cu couples and preserving the sequential interactions among these elements. The effects of Zn concentration on interfacial phase evolution, elemental diffusion, and reaction mechanisms were systematically investigated. For the Sn–1Zn and Sn–3Zn couples, Ag_3_Sn preferentially formed near the solder/Ag interface during the early stage, accompanied by Ag_5_Zn_8_ in the inner reaction region. With prolonged aging, the Ag–Zn phases were progressively redistributed into the solder, while the reaction at the Cu side evolved from Cu_5_Zn_8_ to CuZn, and eventually to Cu_6_Sn_5_ as Zn was progressively depleted. In contrast, the Sn–9Zn couple exhibited a distinct reaction pathway, initially dominated by Ag_5_Zn_8_, AgZn_3_, and Cu_5_Zn_8_, with no continuous Ag_3_Sn layer detected. The higher initial Zn content provided a greater supply of Zn for interfacial reactions, leading to substantial Zn consumption and accelerating the transition from Cu–Zn to Cu–Sn reactions, with localized Cu consumption and subsequent Cu_6_Sn_5_ formation. Comparison with the Sn–Zn/Ag couples, in which AgZn was formed, further demonstrated that the limited Ag supply from the thin Ag layer in the Ag/Cu couples favored Ag_5_Zn_8_ formation and promoted the continuous redistribution of Ag into the solder.

## 1. Introduction

Lead-free solders have attracted considerable attention for electronic packaging applications due to environmental regulations and health concerns regarding the use of Pb-containing solders. Among various lead-free solder systems, the eutectic Sn–9 wt.%Zn alloy is a well-established solder that offers a relatively low melting temperature of 198.5 °C, favorable mechanical properties, and low material cost compared with Sn–Ag–Cu alloys [1,2,3]. In addition to binary Sn–Zn compositions, minor Zn additions have been incorporated into Sn–Ag, Sn–Ag–Cu, and Sn–Bi systems to enhance their mechanical properties; for example, the addition of 1 wt.%Zn to Sn–3.5Ag has been reported to significantly improve its mechanical properties [4,5,6,7].

Furthermore, Zn content strongly affects the microstructure and phase constituents of Sn–Zn solders and thus significantly influences their interfacial reactions with substrates and various surface finishes, such as Cu and ENIG (electroless nickel/immersion gold) [7,8,9,10,11]. During the solid-state reaction between Sn–Zn solders and a Cu substrate, Cu_5_Zn_8_ initially forms at the interface. With continued reaction, the Cu_5_Zn_8_ layer becomes unstable and is gradually consumed, resulting in void formation and subsequent penetration of Sn into the voids, which ultimately leads to substantial local consumption of the Cu substrate [11,12,13]. Therefore, understanding the effect of Zn concentration on interfacial phase formation is essential for evaluating the reliability and applicability of Sn–Zn solders in electronic packaging.

Ag is widely used as a surface-finish material, particularly as an immersion Ag finish, owing to its high electrical conductivity, excellent solderability, and compatibility with electronic packaging processes [14,15,16]. During soldering with Zn-containing solders, the Ag finish layer readily dissolves into the molten solder, and the dissolved Ag subsequently reacts with Sn form Ag_3_Sn upon cooling, while also reacting with Zn to form Ag–Zn intermetallic compounds (IMCs) [17,18,19]. These interfacial reactions can significantly affect the evolution of the interfacial microstructure and the reliability of solder joints. However, most previous studies [18,19,20,21] on Ag surface finishes have typically employed liquid-state reflow to form solder joints. Under such conditions, the molten solder can rapidly dissolve and consume the Ag layer, substantially altering the Ag/solder interface and causing the resulting microstructure to primarily reflect reactions between the solder and the underlying Cu substrate rather than the initial reactions at the Ag/solder interface. Consequently, the effect of Zn concentration on the solid-state interfacial reactions between Sn–Zn solders and Ag is difficult to clearly isolate. A controlled solid-state reaction configuration is therefore useful for preserving the Ag layer and isolating subsequent interfacial reactions. Such solid-state reactions are also of increasing interest because solid-state and direct-bonding approaches are being explored for advanced electronic packaging, where interfacial reactions can occur without the extensive melting and dissolution associated with conventional solder reflow.

In this study, Sn–1Zn, Sn–3Zn, and Sn–9Zn solders, containing 1, 3, and 9 wt.%Zn, respectively, were employed to investigate the effect of Zn concentration on solid-state interfacial reactions in Ag/Cu structures. A ~2 μm thick Ag layer was directly electroplated onto the Sn–Zn solder, followed by deposition of a ~50 μm thick Cu layer to form Sn–Zn/Ag/Cu reaction couples. The couples were aged directly at 170 °C without reflow, allowing the Ag layer to remain at the interface during the initial stage of reaction. The selected Ag thickness provided a finite Ag reservoir for examining the formation and subsequent consumption of Ag-containing IMCs, whereas the substantially thicker Cu layer provided a relatively large Cu reservoir under the present aging conditions. The interfacial microstructural evolution and reaction products were systematically examined at three Zn concentrations and different aging times, with particular emphasis on the formation and evolution of Ag–Sn and Ag–Zn IMCs and their interactions with the Cu layer. Sn–Zn/Ag reaction couples with a thick Ag layer were also prepared as a reference system to provide a substantially greater Ag supply than that available in the Sn–Zn/Ag/Cu couples. This reference configuration further enabled the influence of Ag supply on interfacial phase evolution to be distinguished from the effects of limited Ag availability in the Sn–Zn/Ag/Cu couples. These experiments were designed to clarify how Zn concentration and Ag supply affect the formation and evolution of interfacial IMCs under solid-state aging conditions.

## 2. Materials and Methods

Three Sn–Zn solder alloys, Sn–1Zn, Sn–3Zn, and Sn–9Zn, were prepared from high-purity Sn (99.99 wt.%) and Zn shots (99.99 wt.%). For each Sn–Zn alloy, 5 g of the constituent elements was weighed and sealed in a quartz tube (6 mm inner diameter) under a vacuum of approximately 10^−2^ Torr to prevent oxidation during homogenization. The encapsulated samples were heated at 550 °C for 3 days to promote homogenization and then immediately quenched in cold water. The resulting ingots were ground into square pillars and polished with alumina powder to obtain smooth, flat surfaces. An Ag layer (~1.5 μm) was electroplated onto the polished Sn–Zn pillars at room temperature using a silver cyanide (AgCN) plating bath at a current density of 1 A/dm^2^ (ASD). Subsequently, a Cu layer (~50 μm) was electroplated onto the Ag-coated Sn–Zn specimens using a copper sulfate-based plating bath at 2 ASD. For control experiments, binary Sn–1Zn/Ag, Sn–3Zn/Ag, and Sn–9Zn/Ag diffusion couples were prepared under identical electroplating conditions, with the Ag thickness increased to ~50 μm.

To minimize unintended interfacial reactions between the Sn–Zn alloys and the Ag layer, the as-plated reaction couples were either characterized immediately or subjected to thermal aging. The Sn–Zn/Ag/Cu couples were isothermally aged at 170 °C for predetermined times on a hot plate under ambient atmosphere. The specimens were covered with glass-fiber insulation during aging to reduce heat loss and improve temperature stability. Following aging, the specimens were quenched to room temperature, mounted in epoxy resin, and prepared using standard metallographic grinding and polishing procedures. To clearly reveal the interfacial IMC microstructures, the polished cross-sections were lightly etched using a Sn-selective etching solution. The interfacial microstructures were examined using scanning electron microscopy (SEM, FEI Quanta 3D FEG, Thermo Fisher Scientific, Waltham, MA, USA) in the backscattered electron (BSE) mode. Additionally, interfacial cross-sections were prepared by focused ion beam (FIB) milling and subsequently characterized using ion-induced secondary electron (ISE) imaging. The compositions of the IMCs were quantitatively analyzed using an electron probe microanalyzer (EPMA, JEOL JXA-8530F Plus, Tokyo, Japan) operated by trained technical personnel and properly calibrated prior to analysis. Phase identification was based on the measured compositions, established phase relations, and previously reported compositions of the corresponding IMCs.

## 3. Results and Discussion

**Figure 1:**
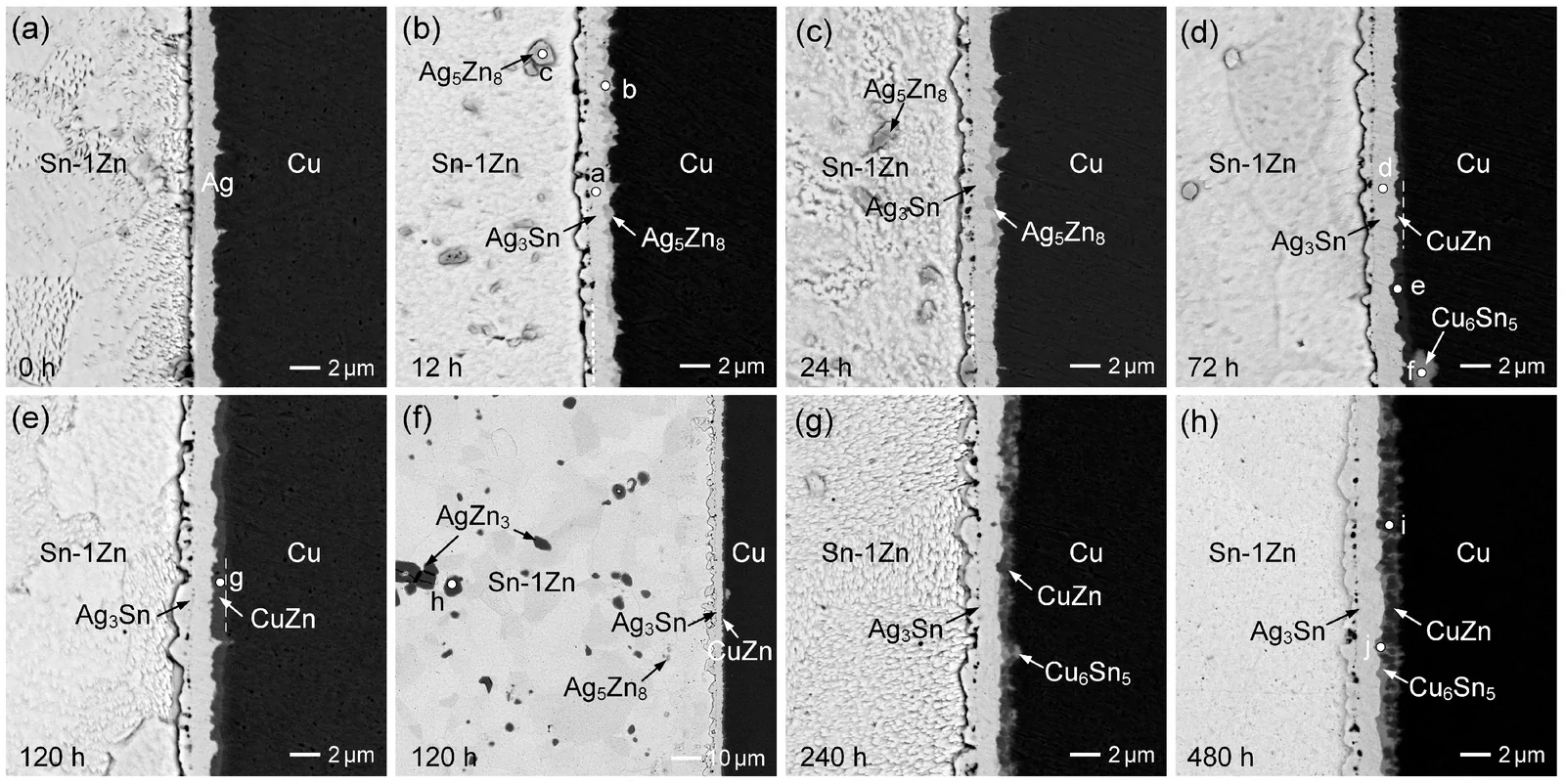
BSE micrographs showing the interfacial microstructural evolution of the Sn–1Zn/Ag/Cu reaction couple during solid-state aging at 170 °C: (**a**) initial state, (**b**) 12 h, (**c**) 24 h, (**d**) 72 h, (**e**) 120 h, (**f**) 120 h at low magnification, (**g**) 240 h, and (**h**) 480 h. Points a–j indicate the locations of EPMA compositional analyses.

The interfacial microstructural evolution of the Sn–1Zn/Ag/Cu couple during solid-state aging at 170 °C is shown in Figure 1. In the as-plated condition (Figure 1a), a ~1.5 μm thick Ag layer was located between the Sn–1Zn solder and Cu substrate. A small amount of reaction product was already present at the Sn–1Zn/Ag interface, suggesting that some interfacial reaction had occurred during sample preparation. After 12 h of aging (Figure 1b), the Ag layer was largely consumed, resulting in the formation of a bright Ag_3_Sn layer (~2 μm) adjacent to the solder and discontinuous dark Ag_5_Zn_8_ regions near the Cu substrate. EPMA analysis revealed the compositions of approximately 70.36 at.%Ag–26.23 at.%Sn–3.41 at.%Zn (point a) and 38.58 at.%Ag–61.05 at.%Zn–0.37 at.%Sn (point b), corresponding to Ag_3_Sn and Ag_5_Zn_8_, respectively. In particular, the Zn content of approximately 3.4 at.% detected in the Ag_3_Sn phase falls within the reported homogeneity range of Ag_3_Sn, consistent with the Sn–Zn–Ag phase diagram [19], as illustrated in Figure S1. The corresponding EPMA compositions of the IMCs identified in the Sn–1Zn/Ag/Cu couples are summarized in Table S1.

Notably, the dark Ag_5_Zn_8_ phase was preferentially distributed near the Ag/Cu interface. The different distributions of the Ag_3_Sn and Ag_5_Zn_8_ can be attributed to concentration gradients and the diffusion of Sn and Zn across the reaction zone. Sn was directly supplied from the solder and preferentially reacted with Ag near the solder side to form Ag_3_Sn. In contrast, despite its lower concentration in the solder, Zn could diffuse through the Ag_3_Sn layer toward the inner region, where it reacted with the remaining Ag under Sn-deficient conditions, leading to the formation of Ag_5_Zn_8_.

With further aging to 24 h (Figure 1c), the interfacial microstructure remained similar to that observed after 12 h. The Ag_3_Sn layer showed no significant change in thickness, whereas the inner Ag_5_Zn_8_ phase gradually disappeared and a Cu-containing reaction layer began to form adjacent to the Cu substrate. A row of regularly distributed fine voids within the Ag_3_Sn layer, as indicated by the dashed lines in Figure 1b,c, was considered to mark the original solder/Ag interface. After 72 h of aging (Figure 1d), Ag_5_Zn_8_ was no longer present, whereas Ag_3_Sn remained approximately 2 μm thick. Meanwhile, a distinct CuZn layer (~0.6 μm thick), with a composition of 51.85 at.%Cu–47.36 at.%Zn–0.13 at.%Ag–0.66 at.%Sn, formed adjacent to the Cu substrate, accompanied by the localized formation of Cu_6_Sn_5_ in a few regions. The formation of the CuZn layer consumed Zn near the Cu interface, which may have facilitated Sn diffusion toward the Cu substrate and the subsequent formation of Cu_6_Sn_5_. These results indicate that, following consumption of the Ag layer, the dominant interfacial reactions gradually shifted from Ag–Sn and Ag–Zn reactions toward Cu–Zn and Cu–Sn reactions.

After 120 h (Figure 1e), the interfacial microstructure remained dominated by Ag_3_Sn on the solder side and CuZn on the Cu side. The corresponding low-magnification image in Figure 1f reveals a substantial number of Ag_5_Zn_8_ and AgZn_3_ particles distributed within the Sn–1Zn solder beyond the continuous interfacial layer, consistent with dispersed Ag_5_Zn_8_ particles present at earlier aging stages (Figure 1b,c). Owing to the strong chemical affinity between Ag and Zn [17], these Ag–Zn IMCs likely formed through the solid-state diffusion of Ag from the original Ag layer into the solder, followed by reactions with Zn. Because no reflow process was involved in this study, their formation can be attributed to solid-state interdiffusion rather than Ag dissolution into molten solder followed by precipitation during cooling.

With further aging to 240 h and 480 h, as displayed in Figure 1g and Figure 1h, respectively, the originally formed CuZn layer slightly thickened to ~1.1 μm and became increasingly heterogeneous, with a large number of fine bright particles distributed within the layer. In contrast, the Ag_3_Sn layer remained continuous and showed essentially no change in thickness. EPMA analysis revealed the compositions of 46.96 at.%Cu–35.45 at.%Zn–3.77 at.%Ag–13.83 at.%Sn for the dark matrix and 51.42 at.%Cu–38.87 at.%Sn–5.32 at.%Zn–4.38 at.%Ag for the bright particles. Based on the Sn–Zn–Cu phase diagram [22], as illustrated in Figure S2, the dark matrix and bright particles were identified as CuZn and Cu_6_Sn_5_, respectively. The CuZn phase exhibits a relatively broad solubility range for Zn and Sn, which is consistent with the measured composition of the dark matrix. Thus, the CuZn layer gradually evolved into a mixed CuZn/Cu_6_Sn_5_ reaction region during prolonged aging. This evolution was associated with progressive Zn depletion, which may have reduced the stability of CuZn and favored the formation of Cu_6_Sn_5_ within the pre-existing CuZn layer. Meanwhile, the Ag_3_Sn layer showed little further growth, likely due to the limited Ag supply from the initially thin Ag layer. The mixed CuZn/Cu_6_Sn_5_ layer also exhibited little further thickening, indicating that the interfacial microstructure approached a relatively stable state.

**Figure 2:**
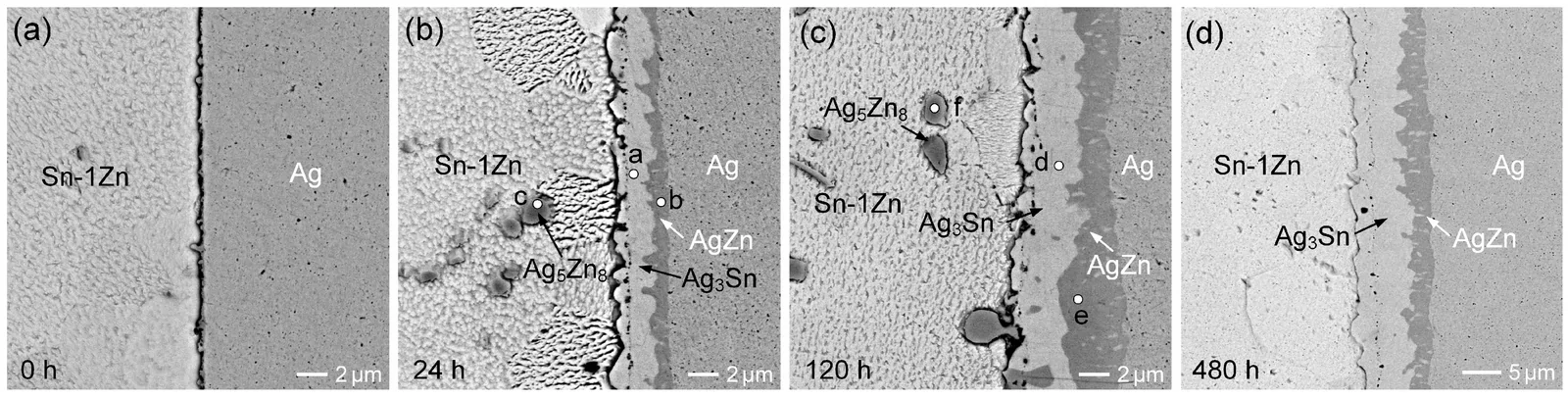
BSE micrographs of the Sn–1Zn/Ag couple during solid-state aging at 170 °C: (**a**) initial state, (**b**) 24 h, (**c**) 120 h, and (**d**) 480 h. Points a–f indicate the EPMA analysis locations.

To further clarify the role of the Cu substrate in the interfacial reaction, a Sn–1Zn/Ag couple without a Cu substrate was also investigated. As shown in Figure 2a, the Ag layer remained intact before aging. After 24 h of aging (Figure 2b), continuous Ag_3_Sn (~2.3 μm) and AgZn (~0.8 μm) layers formed at the Sn–1Zn/Ag interface, accompanied by dispersed Ag_5_Zn_8_ particles in the solder matrix, consistent with the previous study [17]. EPMA analyses at points a–c confirmed the corresponding phase compositions, with the results summarized in Table S2. With further aging for 120 h and 480 h (Figure 2c,d), Ag_3_Sn and AgZn continued to develop as the primary reaction products, with their thicknesses increasing with aging time. The Ag_3_Sn layer increased from ~3.3 μm at 120 h to ~7 μm at 480 h, whereas the AgZn layer showed only a slight increase, from ~2.3 to ~2.5 μm. The Ag_5_Zn_8_ particles remained dispersed throughout the Sn–1Zn solder matrix.

The Ag–Zn IMC phases formed in the Sn–1Zn/Ag and Sn–1Zn/Ag/Cu reaction couples were markedly different. AgZn was predominantly formed at the Sn–1Zn/Ag interface, whereas Ag_5_Zn_8_ was preferentially observed in the inner region of the Sn–1Zn/Ag/Cu couple. This difference can be attributed to different Ag supply conditions in the two configurations. The relatively thin Ag layer in the Sn–1Zn/Ag/Cu couple was rapidly depleted during aging, which favored Ag_5_Zn_8_ formation under limited Ag availability, whereas the thicker Ag layer in the Sn–1Zn/Ag couple provided a greater Ag supply and favored the formation and continued growth of AgZn at the interface.

**Figure 3:**
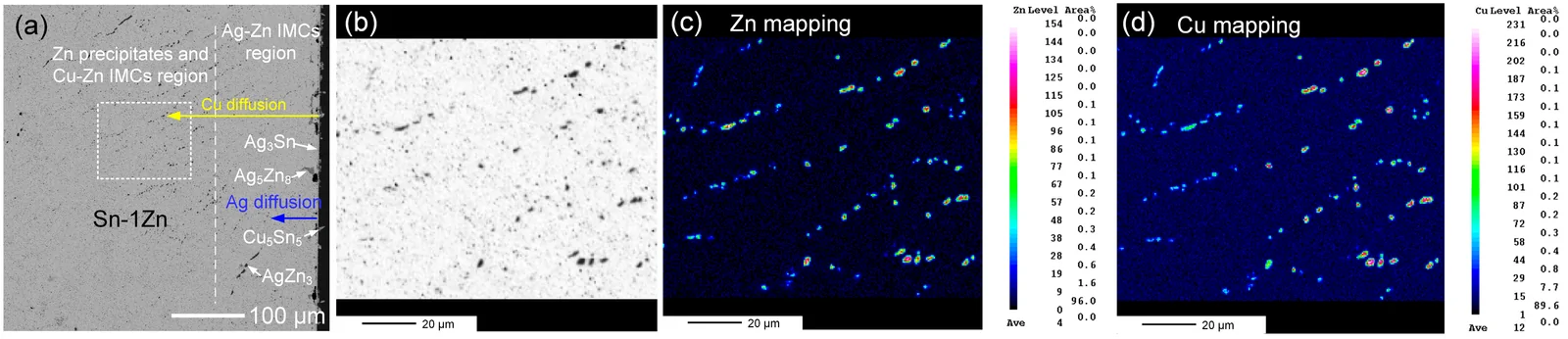
(**a**) BSE micrograph of the Sn–1Zn/Ag/Cu couple after solid-state aging at 170 °C for 72 h. (**b**) Enlarged BSE micrograph of the region enclosed by the white dashed rectangle in (**a**). (**c**) Zn and (**d**) Cu elemental maps obtained by EPMA from the region shown in (**b**).

To further investigate the diffusion behavior of Ag and Cu from the substrates during the interfacial reaction, the corresponding elemental distributions were analyzed. As shown in Figure 3a, the Ag–Zn IMCs, i.e., Ag_5_Zn_8_ and AgZn_3_, were mainly distributed near the original Ag/solder interface, suggesting that Ag diffusion into the solder was relatively limited. In contrast, numerous fine Zn precipitates were observed in the solder region farther from the interface. To further characterize the elemental distributions in this region, Zn and Cu elemental mapping was performed on the area shown in Figure 3b, as presented in Figure 3c and Figure 3d, respectively. The elemental maps clearly show that Cu was distributed over a considerably wider region extending into the Sn–1Zn solder. Fine Cu-containing particles were also detected in regions farther from the interface. Although these particles were too fine to be precisely identified, the spatial overlap of the Cu and Zn signals suggests that they were likely Cu–Zn IMCs. A relatively high Cu concentration was also observed near the Cu/solder interface, indicating continued Cu diffusion from the Cu substrate into the solder.

**Figure 4:**
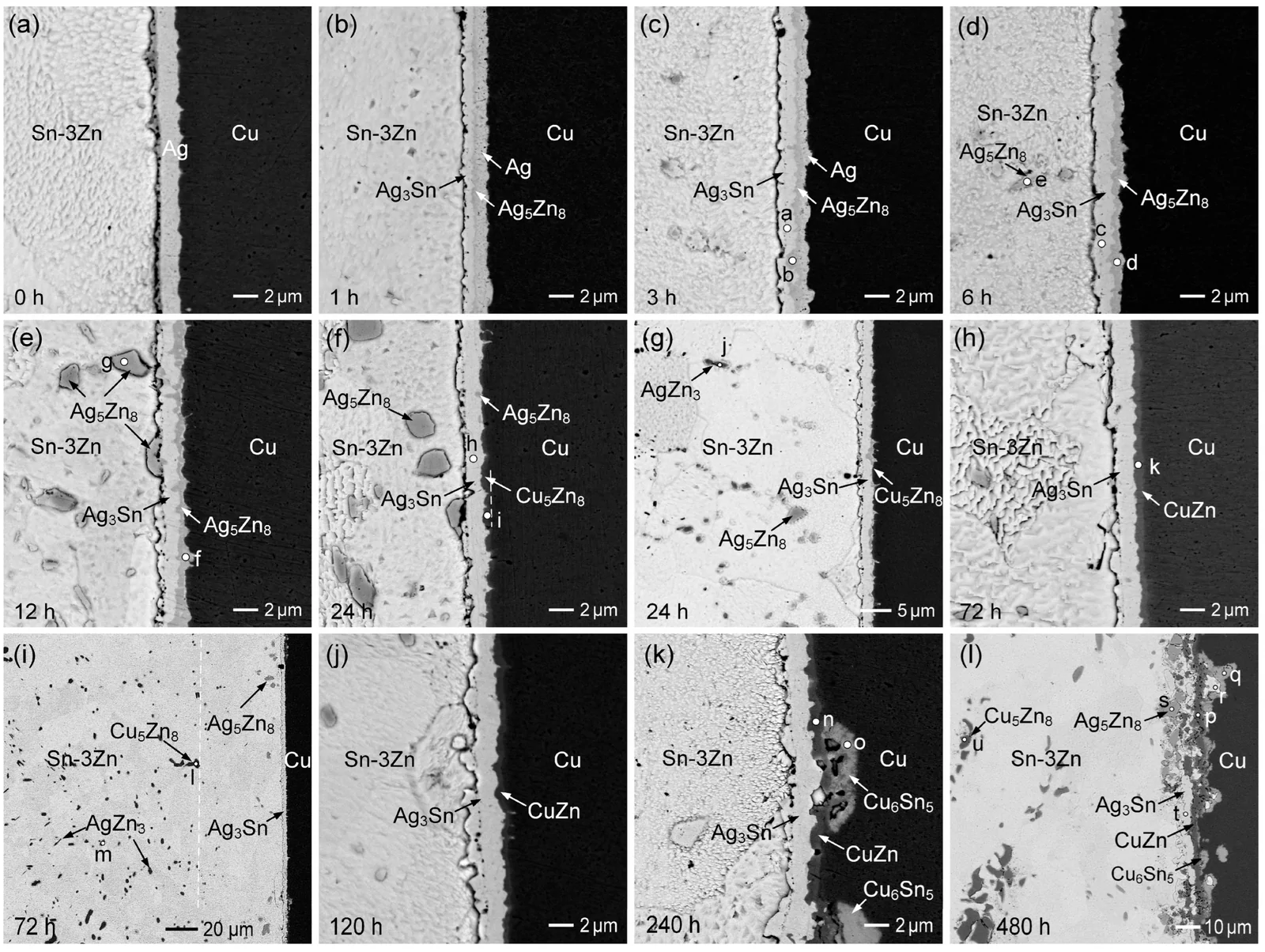
BSE micrographs showing the interfacial microstructural evolution of the Sn–3Zn/Ag/Cu reaction couple during solid-state aging at 170 °C: (**a**) initial state, (**b**) 1 h, (**c**) 3 h, (**d**) 6 h, (**e**) 12 h, (**f**) 24 h, (**g**) 24 h at low magnification, (**h**) 72 h, (**i**) 72 h at low magnification, (**j**) 120 h, (**k**) 240 h, and (**l**) 480 h at low magnification. Points a–u indicate the EPMA analysis locations.

With the Zn content increased to 3 wt.%, the interfacial microstructural evolution of the Sn–3Zn/Ag/Cu couple during aging at 170 °C is shown in Figure 4. Before aging (Figure 4a), granular reaction-phase particles had already formed at the Sn–3Zn/Ag interface after electroplating, similar to those observed in the Sn–1Zn/Ag/Cu couple. To examine the early-stage evolution, the couples were aged for 1, 3, 6, and 12 h (Figure 4b–e). After 1 h of aging, the Ag layer adjacent to the solder was converted into an approximately 0.9 µm thick Ag_3_Sn layer. In addition, a thin continuous dark-contrast IMC layer formed between Ag_3_Sn and the remaining Ag layer, which was approximately 1 μm thick. After 3 h of aging, the remaining Ag layer was almost completely consumed, and the dark-contrast layer became noticeably thicker and was identified as Ag_5_Zn_8_. Discrete Ag-Zn IMC particles were also distributed within the solder. The corresponding EPMA results obtained from points a–u in Figure 4, together with the identified phases in the Sn–3Zn/Ag/Cu couples, are presented in Table S3.

After 6 h of aging (Figure 4d), the original Ag layer was completely consumed, leaving Ag_3_Sn and Ag_5_Zn_8_ as the primary reaction products, with thicknesses of ~1.7 µm and ~0.6 µm, respectively. This reaction sequence was similar to that observed for the Sn–1Zn/Ag/Cu couple, indicating that increasing the Zn content from 1 to 3 wt.% did not substantially alter the initial reaction sequence. After 12 h (Figure 4e), the interfacial microstructure showed little further evolution, while the Ag_5_Zn_8_ particles within the solder matrix became noticeably coarser, likely due to the outward diffusion of Ag from the interface, providing an additional Ag supply for particle growth.

After 24 h of aging (Figure 4f,g), the Ag_3_Sn phase remained relatively stable, with a thickness of ~1.6 μm, whereas the Ag_5_Zn_8_ phase near the Cu substrate was almost completely consumed and replaced by Cu_5_Zn_8_. The Cu_5_Zn_8_ phase exhibited a discontinuous, scallop-like morphology, with a measured composition of 34.11 at.%Cu–64.24 at.%Zn–0.32 at.%Ag–1.33 at.%Sn. In the solder region, dispersed Ag_5_Zn_8_ particles coexisted with the Zn-rich AgZn_3_ phase. With further aging to 72 h (Figure 4h), the continuous Cu–Zn IMC layer (~0.6 μm) became more prominent along the Cu side and was identified as CuZn, with a composition of 52.26 at.%Cu–47.18 at.%Zn–0.13 at.%Ag–0.43 at.%Sn. This transition from Cu_5_Zn_8_ to CuZn was associated with progressive Zn depletion in the reaction region, which favored the formation of the more Zn-deficient CuZn phase. In contrast, the Ag_3_Sn layer remained relatively stable, maintaining a thickness of ~1.7 μm.

As shown in Figure 4i, Ag–Zn IMC particles were distributed throughout the Sn–3Zn solder, with their phase distribution varying with the distance from the interface. Ag_5_Zn_8_ predominated within ~50 μm of the interface, whereas AgZn_3_ became more abundant farther from the interface. The lower density of Ag_5_Zn_8_ particles near the interface may be attributed to continuous Zn diffusion toward the interface, where Zn was gradually consumed by the ongoing interfacial reactions. Additionally, minor Cu_5_Zn_8_ particles were also present within the solder matrix, consistent with Cu diffusion from the substrate into the solder and subsequent reaction with Zn. These results demonstrate that prolonged aging enabled Zn to react with both Ag and Cu supplied from the underlying substrate.

As aging progressed to 120 h (Figure 4j), the CuZn phase continued to develop, although its thickness remained relatively limited, while the Ag_3_Sn layer remained intact near the solder. Similarly, a row of voids within the Ag_3_Sn layer marked the initial solder/Ag contact interface. After 240 h (Figure 4k), the continued depletion of Zn near the interface weakened the Cu–Zn reaction and shifted the reaction toward Cu–Sn, resulting in localized Cu_6_Sn_5_ formation and substantial consumption of the Cu substrate. Unlike a continuous interfacial layer, Cu_6_Sn_5_ developed locally, with Sn-rich regions embedded within the original Cu substrate. Similar localized degradation and interfacial fractures have been reported in Sn–9Zn/Cu couples [11,12]. These features suggest that Cu_6_Sn_5_ formation was associated with outward Cu diffusion from the substrate and inward Sn diffusion toward the Cu substrate. The resulting counter-diffusion led to local redistribution of Cu and Sn, producing Sn-rich regions within the original Cu substrate.

After 480 h, as shown in Figure 4l, the interfacial degradation became much more pronounced compared with that observed after 240 h. Although the Cu substrate had already undergone localized consumption at 240 h, the Ag_3_Sn layer remained continuous, and the interface was relatively intact. After 480 h, however, the Cu substrate was consumed over most regions near the interface, resulting in a highly irregular and disrupted solder/Cu interface. The Ag_3_Sn and CuZn layers also became fragmented and irregular, accompanied by localized Cu_6_Sn_5_ formation, Sn-rich regions within the Cu substrate, and an accumulation of Ag_5_Zn_8_ particles in the solder.

**Figure 5:**
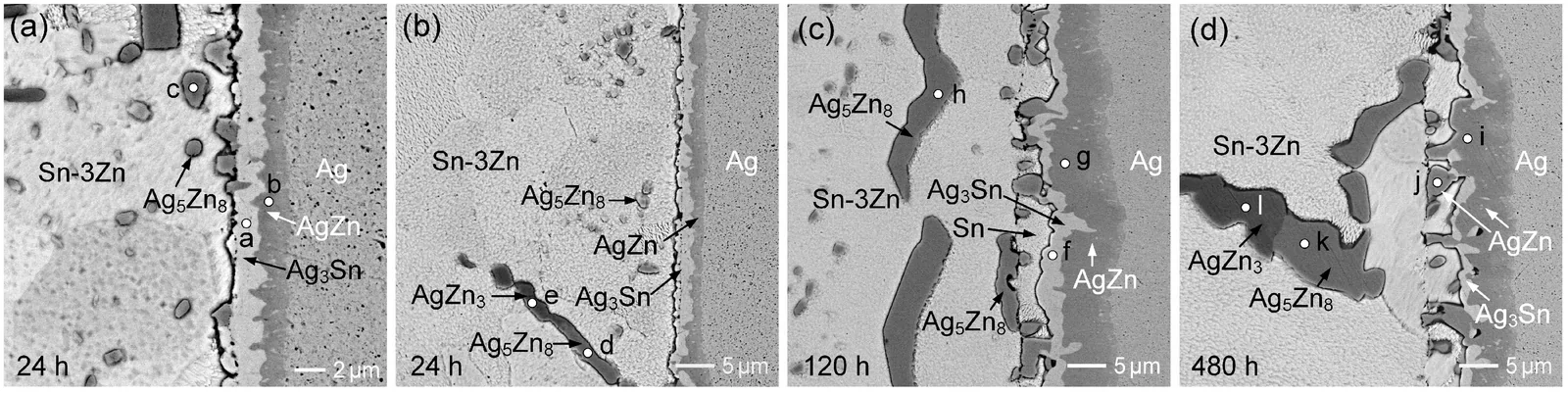
BSE micrographs of the Sn–3Zn/Ag couple during solid-state aging at 170 °C: (**a**) 24 h, (**b**) 24 h at low magnification, (**c**) 120 h, and (**d**) 480 h. Points a–l indicate the EPMA analysis locations.

The Sn–3Zn/Ag reaction couple was investigated for comparison. As displayed in Figure 5a,b, after 24 h of thermal aging, two relatively planar and continuous reaction layers, identified as Ag_3_Sn (~1.7 μm) and AgZn (~1.5 μm), formed adjacent to the Ag substrate, while dispersed Ag_5_Zn_8_ and AgZn_3_ particles were distributed in the solder. The corresponding EPMA results for the Sn-3Zn/Ag reactions obtained from points a–l in Figure 5 are presented in Table S4. In contrast, in the Sn–3Zn/Ag/Cu couple, Ag_5_Zn_8_ preferentially formed near the interface rather than as disperse particles within the solder. This difference can be attributed to the limited Ag supply, which favored the formation of the Zn-richer Ag_5_Zn_8_ phase. A similar dependence of phase selection on the Ag supply was also observed in the Sn–1Zn/Ag/Cu system.

With prolonged aging to 120 h (Figure 5c), the Ag_3_Sn layer showed little thickening (~1.8 μm) and became increasingly irregular, whereas the AgZn phase region became noticeably thicker (~6 μm), accompanied by large Ag_5_Zn_8_ particles dispersed throughout the solder. A narrow etching groove, likely corresponding to the original solder/Ag interface, was observed within the interfacial region. The groove and the adjacent region were filled with Sn-rich material. These features suggest that Ag was continuously redistributed from the Ag substrate into the solder, where it reacted with Zn to form Ag_5_Zn_8_. As the Ag substrate was progressively consumed, the initially planar Ag_3_Sn layer became increasingly irregular and curved. The concurrent dissolution of Ag from Ag_3_Sn into the solder likely offset the continued formation of Ag_3_Sn at the Ag_3_Sn/AgZn interface, resulting in little net thickening of the Ag_3_Sn layer. After 480 h (Figure 5d), the interfacial microstructure remained similar, although the Ag_3_Sn layer became thinner and locally disappeared in some regions, providing further evidence of continued Ag consumption and redistribution.

Based on the above observations, the interfacial reactions can be divided into three stages. During the early stage (≤12 h), rapid interdiffusion among Ag, Sn, and Zn promotes the formation of Ag_3_Sn, with AgZn forming in the Sn–3Zn/Ag couple and Ag_5_Zn_8_ preferentially forming in the Sn–3Zn/Ag/Cu couples. During the intermediate stage (24 h–120 h), Ag_3_Sn in the Sn–3Zn/Ag couple continued Ag redistribution into the solder, where Ag reacts with Zn to form Ag_5_Zn_8_, resulting in limited net growth of Ag_3_Sn. In contrast, the Sn–3Zn/Ag/Cu couple exhibits a transition from Cu_5_Zn_8_ to CuZn as the local Zn content decreases. Upon prolonged aging (240–480 h), further Zn depletion favors Cu_6_Sn_5_ formation, accompanied by localized voiding and Sn penetration into the Cu substrate.

**Figure 6:**
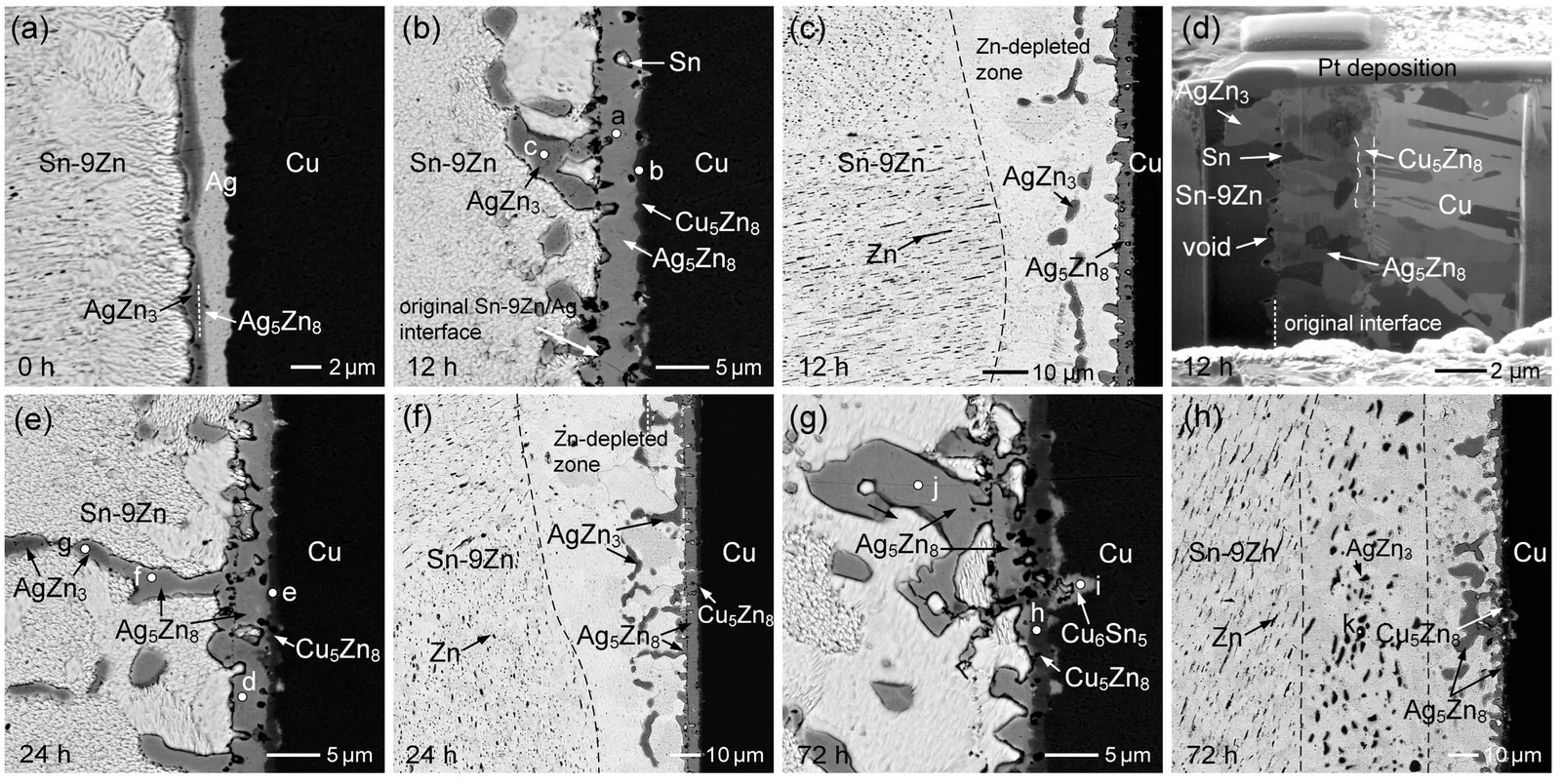
BSE micrographs showing the interfacial microstructural evolution of the Sn–9Zn/Ag/Cu reaction couple during solid-state aging at 170 °C: (**a**) initial state, (**b**) 12 h, (**c**) 12 h at low magnification, (**d**) FIB cross-sectional and ISE images after 12 h, (**e**) 24 h, (**f**) 24 h at low magnification, (**g**) 72 h, and (**h**) 72 h at low magnification. Points a–j indicate the EPMA analysis locations. The black dashed lines indicate the boundary between different characteristic regions of the Sn–Zn solder.

When the Zn concentration was increased to 9 wt%, the interfacial reaction behavior became distinctly different from that of the Sn–1Zn and Sn–3Zn solders. Figure 6a shows the initial microstructure of the Sn–9Zn/Ag/Cu couple after electroplating. A thin Ag–Zn reaction layer was observed at the solder/Ag interface, exhibiting two distinct contrast regions upon closer examination. Based on the compositional analyses at longer aging times, these regions were tentatively identified as AgZn_3_ and Ag_5_Zn_8_, respectively. This observation indicates that interfacial reactions had already initiated during electroplating and continued during the subsequent metallographic preparation period of approximately 10 h.

After aging at 170 °C for 12 h, as shown in Figure 6b, the original Ag layer was completely consumed, resulting in the formation of an Ag–Zn IMC layer approximately 3.5 μm thick. For the Sn–9Zn/Ag/Cu reaction, the compositions of the resulting IMCs were further analyzed by EPMA, with the results summarized in Table S5. The coarse grains distributed within the solder matrix adjacent to the Ag_5_Zn_8_ layer were identified as AgZn_3_. A continuous Cu_5_Zn_8_ layer developed adjacent to the Cu substrate, indicating that the abundant Zn in the Sn–9Zn solder supported simultaneous Ag–Zn and Cu–Zn reactions. Notably, several Sn-rich regions were locally observed within the Ag_5_Zn_8_ layer, suggesting that Sn had diffused into or accumulated within the Ag–Zn reaction layer.

At a lower magnification, as shown in Figure 6c, a large number of Zn-rich precipitates appeared in the Sn–9Zn solder region to the left of the dashed lines, whereas these precipitates were largely absent near the interface, indicating the formation of a Zn-depleted zone. This depletion was associated with the preferential Zn consumption during the interfacial Ag–Zn reaction, with AgZn_3_ grains forming near the reaction layer. Unlike the Sn–1Zn and Sn–3Zn couples, no Ag_3_Sn layer was detected in the Sn–9Zn couple. Instead, the interfacial reaction was dominated by Ag_5_Zn_8_ and Cu_5_Zn_8_, together with dispersed AgZn_3_ grains. This difference suggests that the higher Zn concentration altered the dominant interfacial reaction pathway. To further examine the formation of Cu_5_Zn_8_, FIB cross-sectional observations were performed, as shown in Figure 6d. The Cu_5_Zn_8_ region exhibited a relatively fine-grained microstructure, whereas the Ag_5_Zn_8_ layer consisted of relatively coarse grains. Several voids were also present near the original Sn–9Zn/Ag interface.

With further aging to 24 h, as shown in Figure 6e,f, the overall interfacial microstructure remained similar to that observed after 12 h. Nevertheless, localized consumption of the interfacial Ag_5_Zn_8_ layer became evident as Ag continued to dissolve and diffuse into the solder. Ag_5_Zn_8_ and AgZn_3_ particles were distributed near and farther from the interface, respectively, consistent with the local Zn concentration gradient across the reaction zone. Upon extending the aging time to 72 h (Figure 6g), the interfacial Ag_5_Zn_8_ layer underwent severe localized consumption, resulting in its partial removal and exposing the underlying Cu_5_Zn_8_ layer. The substantial Zn depletion allowed the localized Cu_6_Sn_5_ formation through the direct reaction of Sn with the Cu substrate. Compared with the Sn–3Zn/Ag/Cu system, where no Cu–Sn reaction occurred even after 120 h, the higher Zn content in Sn–9Zn led to more extensive Ag–Zn and Cu–Zn reactions and greater Zn consumption, accelerating the transition from Cu–Zn to Cu–Sn reactions. At a lower magnification (Figure 6h), Ag_5_Zn_8_ and AgZn_3_ dominated the regions near and farther from the interface, respectively, demonstrating the strong influence of local Zn availability on the formation and spatial distribution of Ag–Zn IMCs.

**Figure 7:**
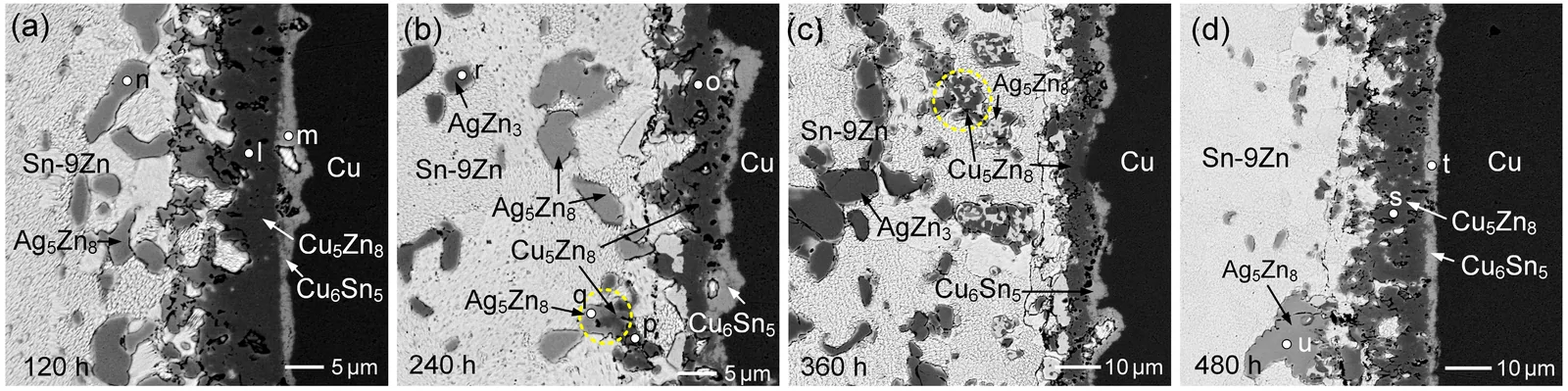
BSE micrographs showing the interfacial microstructural evolution of the Sn–9Zn/Ag/Cu reaction couple during solid-state aging at 170 °C: (**a**) 120 h, (**b**) 240 h, (**c**) 360 h, and (**d**) 480 h. Points l–u indicate the EPMA analysis locations.

With further aging to 120 h (Figure 7a), the interfacial region was mainly composed of Cu_5_Zn_8_ and Cu_6_Sn_5_, as confirmed by the corresponding EPMA analyses summarized in Table S5. The originally continuous Ag_5_Zn_8_ layer had almost completely dissolved and was redistributed as dispersed Ag_5_Zn_8_ particulates within the solder, indicating its interfacial instability. Meanwhile, the Cu_5_Zn_8_ layer continued to thicken, although localized dissolution of Cu_5_Zn_8_ accompanied by void formation was also observed. Although some voids were observed during prolonged aging, no severe or extensive voiding was found within the interfacial IMC layers under the conditions investigated. These observations provide preliminary insight into the extent of interfacial voiding, but its potential effects on solder-joint reliability require further mechanical and electrical evaluation.

After 240 and 360 h of aging (Figure 7b,c), the Ag_5_Zn_8_ layer was further consumed, and some particles containing both Ag_5_Zn_8_- and Cu_5_Zn_8_-rich compositions were observed, as indicated by the yellow dashed circles. According to the Zn–Ag–Cu phase diagram [23] (Figure S3), Ag_5_Zn_8_ and Cu_5_Zn_8_ can form a continuous solid solution, which provides a possible basis for the progressive incorporation of Cu into the Ag5Zn8-rich phase through compositional substitution rather than the formation of a distinct new phase. Therefore, the outward diffusion of Cu from the Cu substrate likely promoted the progressive replacement of Ag by Cu in the Ag–Zn IMCs, resulting in a gradual evolution from Ag_5_Zn_8_-rich to Cu_5_Zn_8_-rich compositions. Upon prolonged aging to 480 h (Figure 7d), the interface remained dominated by Cu_5_Zn_8_ and Cu_6_Sn_5_, with no continuous Ag-containing layer, indicating that the deposited Ag was substantially consumed and redistributed into the solder through the formation and subsequent evolution of Ag–Zn IMCs.

**Figure 8:**
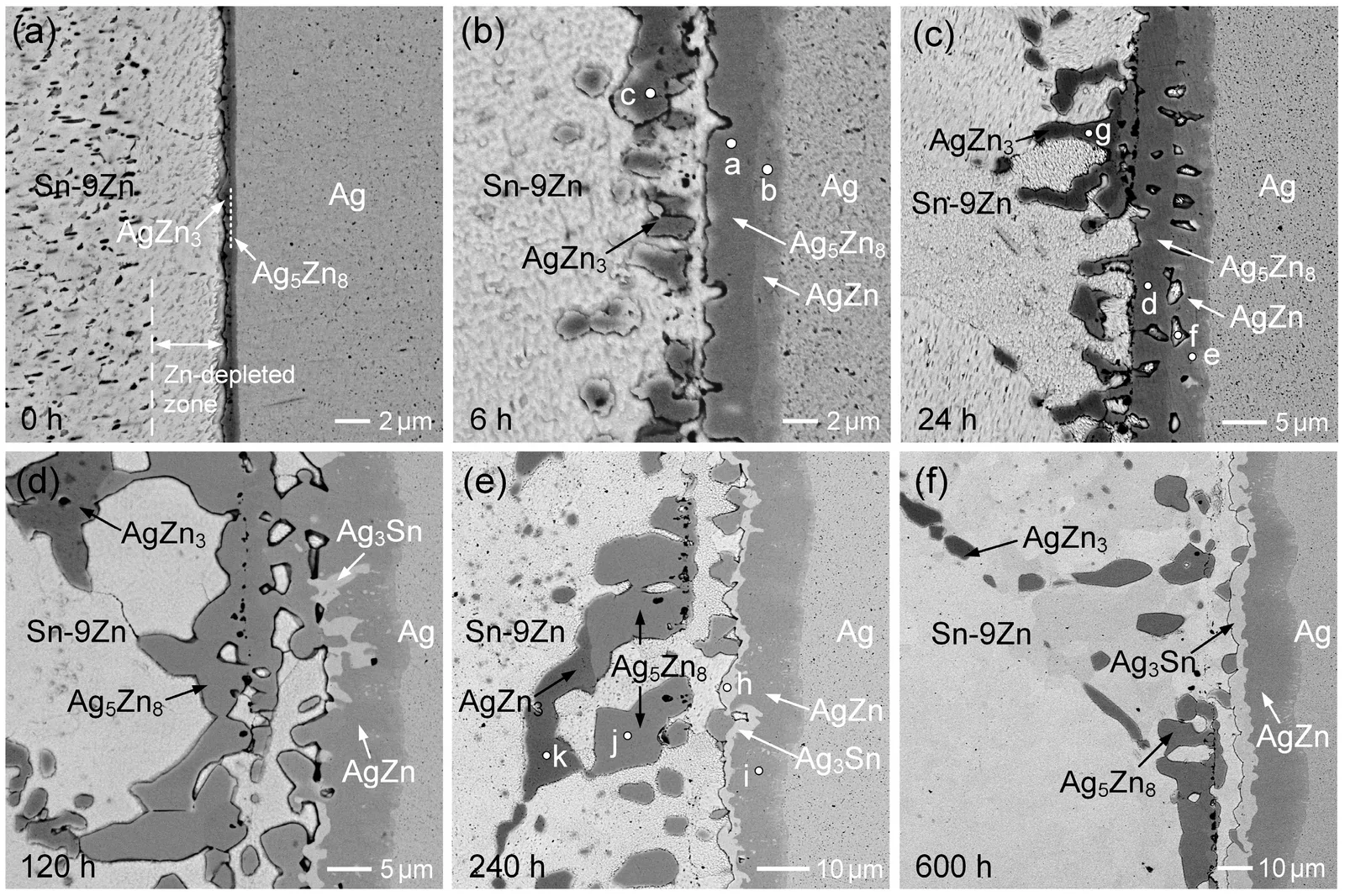
BSE micrographs of the Sn–9Zn/Ag couple during solid-state aging at 170 °C: (**a**) initial state, (**b**) 6 h, (**c**) 24 h, (**d**) 120 h, (**e**) 240 h, and (**f**) 600 h. Points a–k indicate the EPMA analysis locations.

As shown in Figure 8a–f, the interfacial microstructure of the Sn–9Zn/Ag couple evolved progressively during aging at 170 °C. At the initial stage (Figure 8a), similar to the Sn–9Zn/Ag/Cu couple, thin AgZn_3_ and Ag_5_Zn_8_ layers formed at the interface. The marked Zn depletion within ~5 μm of the adjacent solder indicates substantial Zn consumption, suggesting that Zn readily diffused toward the Ag interface and reacted with Ag during the initial stage, including during sample preparation at room temperature. After 6 h of aging, the Ag_5_Zn_8_ and AgZn phases, approximately 2.6 μm and 1.3 μm thick, respectively, formed along the solder/Ag interface, while dispersed AgZn_3_ particles were observed within the solder. The corresponding EPMA results are summarized in Table S6. After 24 h (Figure 8c), these phases remained, while Sn-filled voids emerged within the Ag–Zn IMC layer, indicating local interfacial destabilization and the redistribution of Ag into the solder. After prolonged aging to 120 h (Figure 8d), the Ag–Zn IMC layer became increasingly discontinuous, with a substantial amount of Ag_5_Zn_8_ particulates migrating into the solder. Meanwhile, AgZn became the dominant interfacial phase, with Ag_3_Sn particles embedded within the AgZn layer. At longer aging times of 240 and 600 h, the interfacial microstructure remained similar to that at 120 h, with a continuous Ag_3_Sn layer forming alongside the pre-existing AgZn phase.

**Figure 9:**
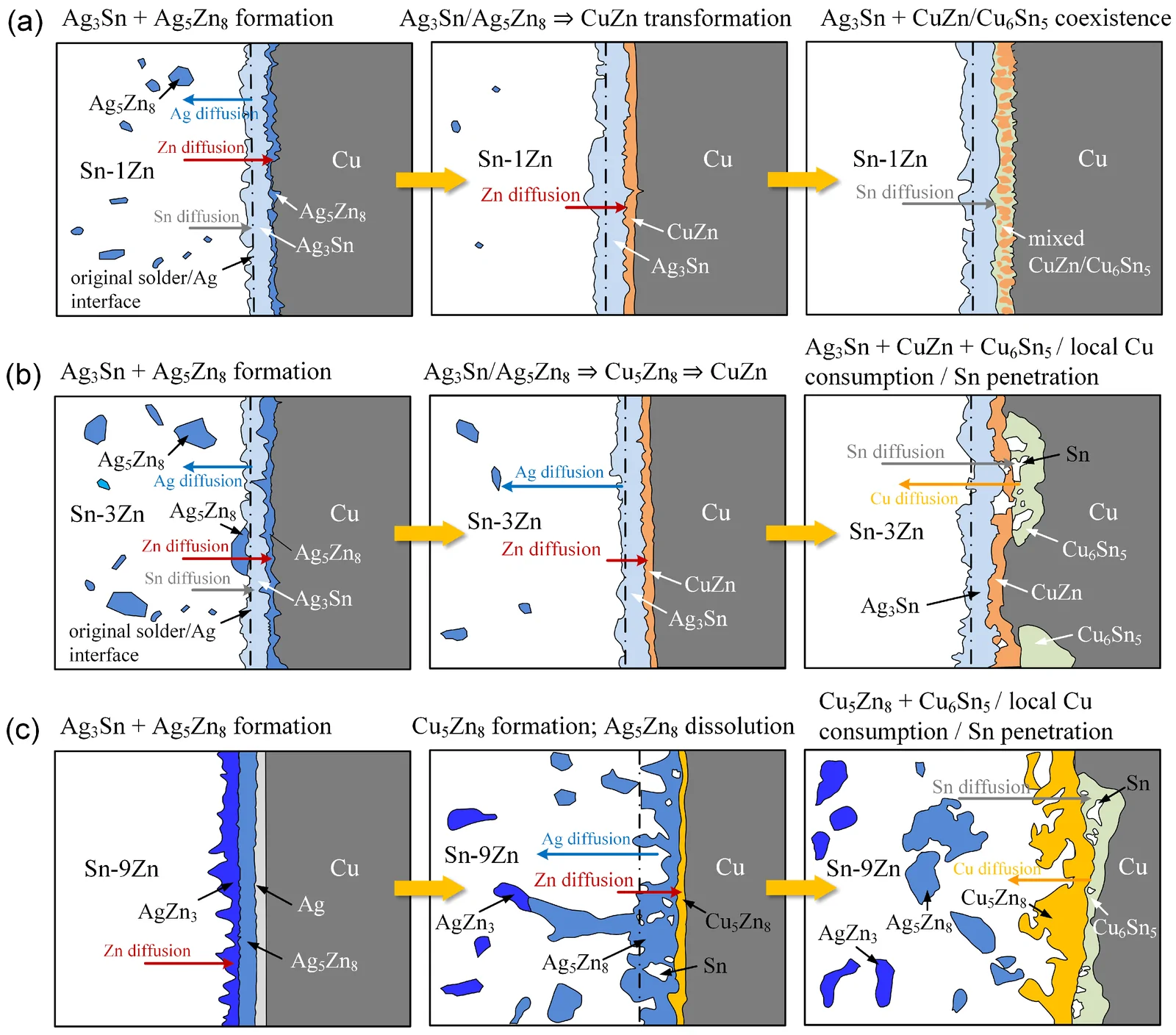
Schematic illustration of the interfacial reaction evolution in Sn–Zn/Ag/Cu couples at 170 °C: (**a**) Sn–1Zn, (**b**) Sn–3Zn, and (**c**) Sn–9Zn.

Systematic examination of the interfacial microstructural evolution revealed that the Sn–Zn/Ag/Cu reaction couples were strongly affected by the Zn concentration in the solder and the available Ag supply. Table 1 summarizes the major interfacial phases and their evolution with aging time in the Sn–1Zn, Sn–3Zn, and Sn–9Zn/Ag/Cu couples, while Figure 9a–c schematically illustrates their corresponding phase evolution. At low Zn contents (1 and 3 wt.%), Ag_3_Sn formed preferentially at the solder/Ag interface during the early stage, accompanied by Ag_5_Zn_8_ in the inner reaction region. With increasing Zn content to 9 wt.%, however, Ag–Zn reactions became dominant, and no continuous Ag_3_Sn layer was detected. Instead, Ag_5_Zn_8_ and AgZn_3_ formed rapidly at the interface and within the solder. These results demonstrate that the local Zn concentration plays an important role in controlling the competition between Ag–Sn and Ag–Zn reactions and, consequently, the selection of Ag-containing IMCs.

Comparison of the Ag-only and Ag/Cu reaction couples further highlights the important role of the Ag supply. In the Sn–1Zn/Ag and Sn–3Zn/Ag couples, the relatively thick Ag layer provided a sufficient Ag reservoir, allowing AgZn to persist as a major interfacial phase during prolonged aging. In contrast, the thin Ag layer in the Ag/Cu couples was rapidly consumed, and the limited Ag supply favored the formation of the Zn-richer Ag_5_Zn_8_ phase. After the original Ag layer was consumed, Ag continued to redistribute from the interfacial reaction zone into the solder, where it reacted with Zn to form dispersed Ag–Zn IMCs. Thus, the final interfacial phase constitution was governed by both the Zn concentration and the local availability and redistribution of Ag.

The observed phase evolution can be explained by the coupled diffusion of Ag, Zn, Sn, and Cu. During the initial stage, Ag diffused from the electroplated layer toward the solder, while Zn and Sn diffused toward the Ag-rich region. Their interdiffusion, driven by the strong chemical affinity of Ag for both Zn and Sn, resulted in the simultaneous formation of Ag–Zn and Ag–Sn IMCs. In the Sn–1Zn and Sn–3Zn systems, Ag_3_Sn preferentially formed near the solder side, whereas Ag_5_Zn_8_ mainly developed in the inner reaction region. This distribution can be attributed to the concentration gradients established across the reaction zone. Sn was continuously supplied from the solder and reacted with Ag near the solder/Ag interface to form Ag_3_Sn. In contrast, Zn could diffuse through the developing Ag–Sn reaction region and react with the remaining Ag under relatively Zn-rich and Sn-deficient conditions, favoring the formation of Ag_5_Zn_8_.

Once the Ag layer was substantially consumed, the interfacial reactions became increasingly dominated by diffusion and reactions involving the Cu substrate. Zn continued to diffuse toward the interface and react with Cu, initially forming the Zn-rich Cu_5_Zn_8_. In the Sn–3Zn couple, Cu_5_Zn_8_ gradually transformed into the Zn-deficient CuZn phase as the local Zn concentration decreased. In contrast, in the Sn–9Zn couple, progressive Zn depletion destabilized the Cu_5_Zn_8_ layer, leading to its partial dissolution and the formation of interfacial voids. Cu subsequently diffused outward from the substrate, while Sn diffused inward toward the Cu substrate and reacted with Cu to form Cu_6_Sn_5_, accompanied by substantial localized consumption of the Cu substrate. Thus, the interfacial reaction evolved from an initial Ag–Sn/Ag–Zn-dominated regime to Cu–Zn reactions and eventually to Cu–Sn reactions. This transition occurred more rapidly with increasing Zn content, consistent with the greater extent of Zn consumption and redistribution at the interface. Compared with previous liquid-state Sn–Zn/Ag reactions [18,19,20,21], the present solid-state aging results demonstrate that Zn concentration and Ag supply strongly influence the interfacial reaction sequence. These findings provide useful insight into the effects of Zn concentration and Ag supply on solid-state interfacial reactions in Sn–Zn lead-free solder systems with Ag surface finishes.

## 4. Conclusions

The solid-state interfacial reactions of Sn–1Zn, Sn–3Zn, and Sn–9Zn with Ag/Cu substrates were systematically investigated at 170 °C to clarify the effects of Zn concentration on phase formation, elemental redistribution, and interfacial evolution. The Ag layer retained between the solder and Cu substrate enabled direct observation of the sequential interactions among Sn, Zn, Ag, and Cu during prolonged solid-state aging. Comparisons among different Zn concentrations and between the Sn–Zn/Ag/Cu and Sn–Zn/Ag couples further highlights the roles of Zn availability and Ag supply in governing the interfacial reaction pathway. The following conclusions can be drawn based on the above results:

1. The interfacial reaction behavior was strongly dependent on Zn concentration and Ag availability. The thin Ag layer acted as an active reaction source rather than simply serving as a surface finish. As aging proceeded, Ag was progressively consumed and redistributed into the solder, sustaining Ag–Zn IMC formation away from the original Ag layer.
2. Increasing the Zn content progressively shifted the Ag-containing reactions from Ag–Sn toward Ag–Zn IMC formation. In the Sn–1Zn and Sn–3Zn couples, Ag_3_Sn formed together with Ag_5_Zn_8_ during the early stage, whereas the reaction in the Sn–9Zn couple was dominated by Ag_5_Zn_8_ and AgZn_3_, with no continuous Ag_3_Sn layer detected. These results indicate that increased Zn availability favored Ag–Zn reactions over Ag–Sn reactions.
3. The reaction at the Cu side was strongly influenced by progressive Zn consumption. Cu_5_Zn_8_ initially formed adjacent to the Cu substrate and subsequently evolved toward the more Zn-deficient CuZn phase. With further aging, continued Zn depletion destabilized the Cu–Zn reaction region and favored the formation of Cu_6_Sn_5_ through the reaction of Sn with the Cu substrate. In the Sn–3Zn and Sn–9Zn couples, this transition was accompanied by localized Cu consumption and interfacial void formation.
4. Comparison with the Sn–Zn/Ag couples demonstrated that the Ag supply condition significantly affected Ag–Zn phase selection. The limited Ag supply in the Ag/Cu couples favored Ag_5_Zn_8_ formation and Ag redistribution into the solder, whereas the thicker Ag source in the Sn–Zn/Ag couples favored AgZn formation adjacent to the Ag substrate. These results demonstrate that both Zn availability and Ag supply are important factors governing the selection and evolution of Ag–Zn IMCs.

## Figures and Tables

**Table 1:** Summary of the major interfacial phases and reaction evolution in the Sn–Zn/Ag/Cu couples with different Zn concentrations during solid-state aging at 170 °C.

Interfacial System	Early Stage (≤24 h)	Intermediate Evolution (72–120 h)	Prolonged Aging (≥240 h)
Sn–1Zn/Ag/Cu	Ag_3_Sn and Ag_5_Zn_8_ form	Ag_3_Sn remains stable; Ag_5_Zn_8_ disappears; CuZn forms	Ag_3_Sn remains stable; mixed CuZn + Cu_6_Sn_5_ layer develops; interface remains relatively stable
Sn–3Zn/Ag/Cu	Ag_3_Sn and Ag_5_Zn_8_ form	Ag_3_Sn remains stable; Ag_5_Zn_8_ disappears; Cu_5_Zn_8_ transforms into CuZn	Ag_3_Sn and CuZn remain stable; Cu_6_Sn_5_ forms with localized Cu consumption and Sn penetration
Sn–9Zn/Ag/Cu	AgZn_3_ and Ag_5_Zn_8_ form	Cu_5_Zn_8_ forms; Ag_5_Zn_8_ dissolves	Cu_5_Zn_8_ remains; Cu_6_Sn_5_ forms with localized Cu consumption and Sn penetration; the Ag–Zn layer nearly disappears

## Data Availability

The original contributions presented in this study are included in the article/Supplementary Material. Further inquiries can be directed to the corresponding author.

## Supplementary Materials

The following supporting information can be downloaded at: https://www.mdpi.com/article/10.3390/ma19194217/s1, Figure S1: The suggested Sn–Zn–Ag phase diagram at 170 °C, estimated from the 260 °C isothermal section reported in Ref. [19]; Figure S2: Isothermal section of the Sn-Zn-Cu phase diagram at 180 °C, reported in Ref. [22]; Figure S3: Isothermal section of the Ag-Cu-Zn phase diagram at 600 °C, reported in Ref. [23]; Table S1: EPMA compositions and phase identification of the Sn-1Zn/Ag/Cu interfacial reactions at 170 °C; Table S2: EPMA compositions and phase identification of the Sn-1Zn/Ag interfacial reactions at 170 °C; Table S3: EPMA compositions and phase identification of the Sn-3Zn/Ag/Cu interfacial reactions at 170 °C; Table S4: EPMA compositions and phase identification of the Sn-3Zn/Ag interfacial reactions at 170 °C; Table S5: EPMA compositions and phase identification of the Sn-9Zn/Ag/Cu interfacial reactions at 170 °C; Table S6: EPMA compositions and phase identification of the Sn-9Zn/Ag interfacial reactions at 170 °C.
