# Supplementary material for: Microstructural Evolution in Solid-State Sn–Zn/Ag/Cu Interfacial Reactions at 170 °C
Source: Materials (Basel). 2026 Oct 3;19(19):4217. doi: 10.3390/ma19194217 (PMC13666448; doi:10.3390/ma19194217)
Supplement: Supplementary file 1 [file materials-19-04217-s001.zip › materials-4574017-supplementary.pdf]

materials

Supplementary material

Microstructural Evolution in Solid-State Sn–Zn/Ag/Cu Interfacial Reactions at 170 ^{o}C

Table S1. EPMA compositions and phase identification of the Sn-1Zn/Ag/Cu interfacial reactions at 170 ^{o}C.

Figure / point	EPMA composition (at.%)	Phase identification
Fig. 1(b) / a	70.36 %Ag–26.23 %Sn–3.41 %Zn	Ag_3Sn
Fig. 1(b) / b	38.58 %Ag–61.05 %Zn–0.37 %Sn	Ag_5Zn_8
Fig. 1(b) / c	36.57 %Ag–62.84 %Zn–0.59 %Sn	Ag_5Zn_8
Fig. 1(d) / d	70.42 %Ag–25.85 %Sn–3.73 %Zn	Ag_3Sn
Fig. 1(d) / e	51.85 %Cu–47.36 %Zn–0.13 %Ag–0.66 %Sn	CuZn
Fig. 1(d) / f	54.92 %Cu–42.65 %Sn–2.37 %Zn–0.06 %Ag	Cu_6Sn_5
Fig. 1(e) / g	50.22 %Cu–46.83 %Zn–0.24 %Ag–2.71 %Sn	CuZn
Fig. 1(f) / h	24.57 %Ag–74.82 %Zn–0.61 %Sn	$AgZn_3$
Fig. 1(h) / i	46.96 %Cu–35.45 %Zn–3.77 %Ag–13.83 %Sn	CuZn
Fig. 1(h) / j	51.42 %Cu–38.88 %Sn–5.32 %Zn–4.38 %Ag	Cu_6Sn_5

Table S2. EPMA compositions and phase identification of the Sn-1Zn/Ag interfacial reactions at 170 ^{o}C.

Figure / point	EPMA composition (at.%)	Phase identification
Fig. 2(b) / a	72.92 %Ag–24.82 %Sn–2.26 %Zn	Ag_3Sn
Fig. 2(b) / b	50.93 %Ag–47.64 %Zn–1.43 %Sn	AgZn
Fig. 2(b) / c	38.96 %Ag–60.56 %Zn–0.48 %Sn	Ag_5Zn_8
Fig. 2(c) / d	71.91 %Ag–25.31 %Sn–2.78 %Zn	Ag_3Sn
Fig. 2(c) / e	49.34 %Zn–48.05 %Ag–2.61 %Sn	AgZn
Fig. 2(c) / f	38.33 %Ag–60.93 %Zn–0.74 %Sn	Ag_5Zn_8

Table S3. EPMA compositions and phase identification of the Sn-3Zn/Ag/Cu interfacial reactions at 170 °C.

Figure / point	EPMA composition (at.%)	Phase identification
Fig. 4(c) / a	70.61 %Ag–24.86 %Sn–4.53 %Zn	Ag_3Sn
Fig. 4(c) / b	40.67 %Ag–56.86 %Zn–2.47 %Sn	Ag_5Zn_8
Fig. 4(d) / c	71.03 %Ag–25.11 %Sn–3.86 %Zn	Ag_3Sn
Fig. 4(d) / d	39.85 %Ag– 57.48%Zn–1.64 %Sn–1.03 %Cu	Ag_5Zn_8
Fig. 4(d) / e	40.16 %Ag–55.71 %Zn–4.13 %Sn	Ag_5Zn_8
Fig. 4(e) / f	39.49 %Ag–56.63 %Zn–2.27 %Sn–1.61 %Cu	Ag_5Zn_8
Fig. 4(e) / g	37.25 %Ag–60.71 %Zn–1.58 %Sn–0.46 %Cu	Ag_5Zn_8
Fig. 4(f) / h	72.28 %Ag–23.94 %Sn–3.51 %Zn–0.27 %Cu	Ag_3Sn
Fig. 4(f) / i	34.11 %Cu–64.24 %Zn–0.32 %Ag–1.33%Sn	Cu_5Zn_8
Fig. 4(g) / j	19.67 %Ag–74.48 %Zn–3.54 %Sn–2.31 %Cu	$AgZn_3$
Fig. 4(h) / k	52.26 %Cu–47.18 %Zn–0.13 %Ag–0.43 %Sn	CuZn
Fig. 4(i) / l	29.76 %Cu–66.34 %Zn–1.72 %Ag–2.18%Sn	Cu_5Zn_8
Fig. 4(i) / m	12.46 %Ag–84.27 %Zn–2.62 %Sn–0.65 %Cu	$AgZn_3$
Fig. 4(k) / n	49.70 %Cu–45.22 %Zn–0.81 %Ag–4.27 %Sn	CuZn
Fig. 4(k) / o	54.61 %Cu–41.15 %Sn–3.38 %Zn–0.86 %Ag	Cu_6Sn_5
Fig. 4(l) / p	47.26 %Cu–39.62 %Zn–10.27 %Sn–2.85 %Ag	CuZn
Fig. 4(l) / q	53.52 %Cu–42.46 %Sn–0.73 %Ag–3.29 %Zn	Cu_6Sn_5
Fig. 4(l) / r	97.44 %Sn–0.23 %Ag–1.48 %Cu–0.85 %Zn	(Sn)
Fig. 4(l) / s	36.15 %Ag–58.76 %Zn–3.47 %Sn–1.62 %Cu	Ag_5Zn_8
Fig. 4(l) / t	70.98 %Ag–25.91 %Sn–2.15 %Zn–0.96 %Cu	Ag_3Sn
Fig. 4(l) / u	40.22 %Cu–56.53 %Zn–0.51 %Ag–2.74%Sn	Cu_5Zn_8

Table S4. EPMA compositions and phase identification of the Sn-3Zn/Ag interfacial reactions at 170 °C.

Figure / point	EPMA composition (at.%)	Phase identification
Fig. 5(a) / a	71.54 %Ag–26.53 %Sn–1.93 %Zn	Ag_3Sn
Fig. 5(a) / b	57.26 %Ag–41.82 %Zn–0.92 %Sn	AgZn
Fig. 5(a) / c	39.17 %Ag–60.18 %Zn–0.65 %Sn	Ag_5Zn_8
Fig. 5(b) / d	37.70 %Ag–61.24 %Zn–1.06 %Sn	Ag_5Zn_8
Fig. 5(b) / e	12.71 %Ag–85.12 %Zn–2.17 %Sn	$AgZn_3$
Fig. 5(c) / f	72.58 %Ag–24.80 %Sn–2.62 %Zn	Ag_3Sn
Fig. 5(c) / g	60.35 %Ag–36.93 %Zn–2.72 %Sn	AgZn
Fig. 5(c) / h	38.99 %Ag–60.18 %Zn–0.83 %Sn	Ag_5Zn_8
Fig. 5(d) / i	52.38 %Ag–45.15 %Zn–2.47 %Sn	AgZn
Fig. 5(d) / j	50.41 %Zn–46.93 %Ag–2.66 %Sn	AgZn
Fig. 5(d) / k	39.92 %Ag–59.30 %Zn–0.78 %Sn	Ag_5Zn_8
Fig. 5(d) / l	14.29 %Ag–83.82 %Zn–1.89 %Sn	$AgZn_3$

Table S5. EPMA compositions and phase identification of the Sn-9Zn/Ag/Cu interfacial reactions at 170 °C.

Figure / point	EPMA composition (at.%)	Phase identification
Fig. 6(b) / a	36.96 %Ag–62.49 %Zn–0.55 %Sn	Ag_5Zn_8
Fig. 6(b) / b	35.83 %Cu–63.05 %Zn–0.39 %Ag–0.73 %Sn	Cu_5Zn_8
Fig. 6(b) / c	23.77 %Ag–75.41 %Zn–0.82 %Sn	$AgZn_3$
Fig. 6(e) / d	39.45 %Ag–58.37 %Zn–1.26 %Sn–0.92 %Cu	Ag_5Zn_8
Fig. 6(e) / e	34.62 %Cu–64.12 %Zn–0.41 %Ag–0.85 %Sn	Cu_5Zn_8
Fig. 6(e) / f	38.07 %Ag–60.57 %Zn–0.94 %Sn–0.42 %Cu	Ag_5Zn_8
Fig. 6(e) / g	15.96 %Ag–82.16 %Zn–1.05 %Sn-0.83 %Cu	$AgZn_3$
Fig. 6(g) / h	36.32 %Cu–61.67 %Zn–0.53 %Ag–1.48 %Sn	Cu_5Zn_8
Fig. 6(g) / i	54.46 %Cu–42.37 %Sn–2.81 %Zn–0.36 %Ag	Cu_6Sn_5
Fig. 6(g) / j	37.50 %Ag–61.03 %Zn–0.82 %Sn–0.65 %Cu	Ag_5Zn_8
Fig. 6(h) / k	11.76 %Ag–86.42 %Zn–0.95 %Sn-0.87 %Cu	$AgZn_3$
Fig. 7(a) / l	35.06 %Cu–63.86 %Zn–0.35 %Ag–0.73 %Sn	Cu_5Zn_8
Fig. 7(a) / m	56.58 %Cu–32.46 %Sn–10.65 %Zn–0.31 %Ag	Cu_6Sn_5
Fig. 7(a) / n	30.90 %Ag–63.91 %Zn–0.43 %Sn–4.76 %Cu	Ag_5Zn_8
Fig. 7(b) / o	34.71 %Cu–63.80 %Zn–0.43 %Ag–1.06%Sn	Cu_5Zn_8
Fig. 7(b) / p	39.16 %Cu–57.46 %Zn–0.91 %Ag–2.47%Sn	Cu_5Zn_8
Fig. 7(b) / q	36.31 %Ag–60.38 %Zn–1.56 %Sn–1.75 %Cu	Ag_5Zn_8
Fig. 7(b) / r	16.08 %Ag–82.25 %Zn–0.74 %Sn-0.93 %Cu	$AgZn_3$
Fig. 7(d) / s	39.48 %Cu–58.37 %Zn–0.47 %Ag–1.68%Sn	Cu_5Zn_8
Fig. 7(d) / t	53.15 %Cu–43.56 %Sn–0.35 %Ag–2.94 %Zn	Cu_6Sn_5
Fig. 7(d) / u	36.20 %Ag–59.52 %Zn–0.66 %Sn–3.62 %Cu	Ag_5Zn_8

Table S6. EPMA compositions and phase identification of the Sn-9Zn/Ag interfacial reactions at 170 °C.

Figure / point	EPMA composition (at.%)	Phase identification
Fig. 8(b) / a	39.58 %Ag–60.08 %Zn–0.34 %Sn	Ag_5Zn_8
Fig. 8(b) / b	51.07 %Ag–48.46 %Zn–0.47 %Sn	AgZn
Fig. 8(b) / c	24.58 %Ag–75.26 %Zn–0.16 %Sn	$AgZn_3$
Fig. 8(c) / d	40.31 %Ag–58.77 %Zn–0.92 %Sn	Ag_5Zn_8
Fig. 8(c) / e	52.15 %Ag–45.11 %Zn–2.74 %Sn	AgZn
Fig. 8(c) / f	97.38 %Sn–0.53 %Ag–2.09 %Zn	(Sn)
Fig. 8(c) / g	20.05 %Ag–79.68 %Zn–0.27 %Sn	$AgZn_3$
Fig. 8(e) / h	72.14 %Ag–25.51 %Sn–2.35 %Zn	Ag_3Sn
Fig. 8(e) / i	47.57 %Ag–51.63 %Zn–0.80 %Sn	AgZn
Fig. 8(e) / j	36.28 %Ag–63.24 %Zn–0.48 %Sn	Ag_5Zn_8
Fig. 8(e) / k	25.71 %Ag–74.17 %Zn–0.12 %Sn	$AgZn_3$

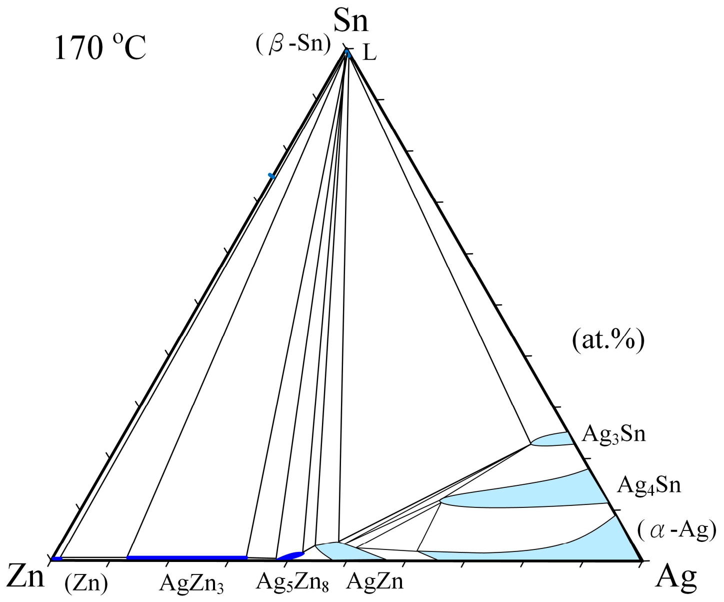

Figure S1. The suggested Sn–Zn–Ag phase diagram at 170 °C, estimated from the 260 °C isothermal section reported in Ref. [19].

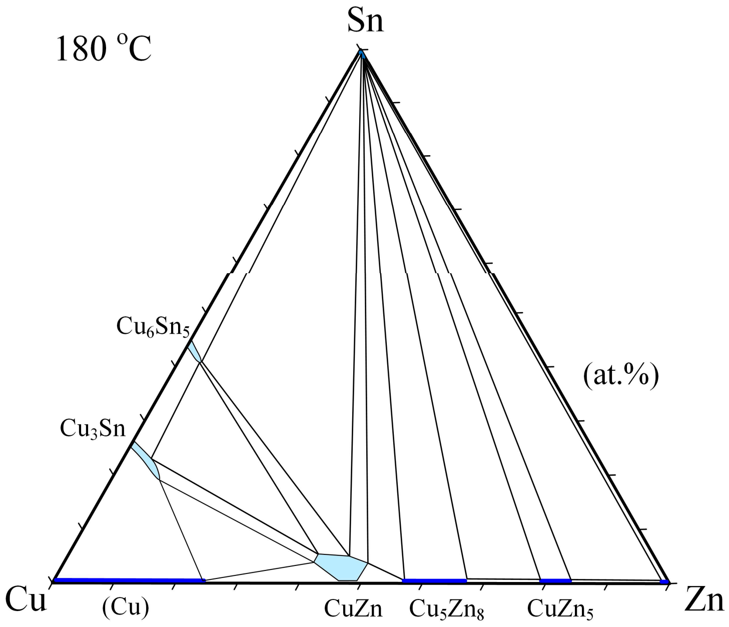

Figure S2. Isothermal section of the Sn-Zn-Cu phase diagram at 180 °C, reported in Ref. [22].

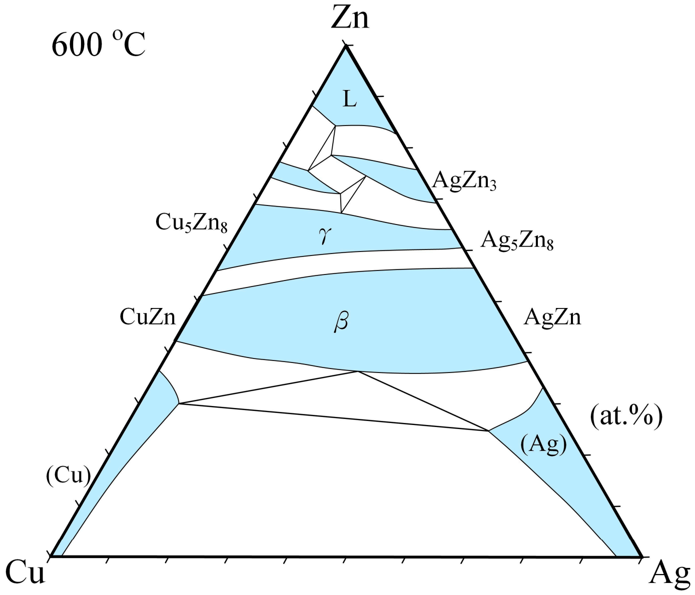

Figure S3. Isothermal section of the Ag-Cu-Zn phase diagram at 600 °C, reported in Ref. [23].
